# Thermodynamic holography

**DOI:** 10.1038/srep15077

**Published:** 2015-10-19

**Authors:** Bo-Bo Wei, Zhan-Feng Jiang, Ren-Bao Liu

**Affiliations:** 1 Department of Physics and Centre for Quantum Coherence, The Chinese University of Hong Kong, Hong Kong, China; 2 Institute of Theoretical Physics, The Chinese University of Hong Kong, Hong Kong, China

## Abstract

The holographic principle states that the information about a volume of a system is encoded on the boundary surface of the volume. Holography appears in many branches of physics, such as optics, electromagnetism, many-body physics, quantum gravity, and string theory. Here we show that holography is also an underlying principle in thermodynamics, a most important foundation of physics. The thermodynamics of a system is fully determined by its partition function. We prove that the partition function of a finite but arbitrarily large system is an analytic function on the complex plane of physical parameters, and therefore the partition function in a region on the complex plane is uniquely determined by its values along the boundary. The thermodynamic holography has applications in studying thermodynamics of nano-scale systems (such as molecule engines, nano-generators and macromolecules) and provides a new approach to many-body physics.

The most famous example of holography is probably the optical hologram, where the three-dimensional view of an object is recorded in a two-dimensional graph^1^. The holographic principle indeed has profound implications in many branches of physics. In electromagnetism, for instance, the electrostatic potential in a volume is uniquely determined by its values at the surface boundary^2^. Density functional theory^3^,^4^, which is the foundation of quantum chemistry and first-principle calculations^4^, may be viewed as a holography that maps the full ground-state wave function of a many-electron system (a complex function in an enormously high-dimensional space) to the ground state single-particle density (a real function in three-dimensional space). The holographic principle has also been shown relevant in quantum gravity^5^ and string theory^6^,^7^. Recently, the holographic approach has been employed to tackle strongly-correlated systems in condensed matter physics^8^.

The physical properties of a system at thermodynamic equilibrium are fully determined by the partition function 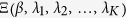 as a function of coupling parameters 
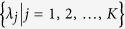
 and the temperature *T* (or the inverse temperature *β* ≡ 1/*T*). In this paper we set the Boltzmann constant *k*_*B*_ and the Planck constant *ħ* to be unity. The partition function is the summation of the Boltzmann factor 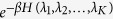 over all energy eigen states, i.e., 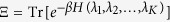, where the Hamiltonian 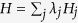 is characterized by a set of coupling parameters {*λ*_*j*_} (e.g., in spin models the magnetic field *h* = *λ*_1_, the nearest neighbor coupling *J* = *λ*_2_, and the next nearest neighbor coupling *J*′ = *λ*_3_, etc.). The normalized Boltzmann factor 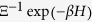 is the probability of the system in a state with energy *H*. In the following we consider one of the physical parameters of the system, 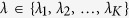 and suppress the other parameters for the simplicity of notation. We assume that the Hamiltonian is a linear function of the parameter, but the main results (the theorems and the corollaries) in this paper apply to Hamiltonians that are general analytic functions of the parameters (see Methods for discussion).

In this article, we establish that the holographic principle holds in thermodynamics, for a finite but arbitrarily large system. We prove that the partition function of a finite physical system is an analytic function of all the physical parameters on the complex plane, and, according to Cauchy theorem^9^, the partition function in any region on the complex plane of a physical parameter is uniquely determined by its values along the boundary. Since the partition function with a complex parameter is equivalent to the coherence of a quantum probe^10^,^11^,^12^, it is physically feasible to determine the whole thermodynamic properties of a system by measuring the probe spin coherence for just one value of the parameter. We theoretically study an experimentally realizable system, namely, a nitrogen-vacancy centre coupled to a mechanical resonator, to demonstrate that the free energy of the resonator is fully determined by the nitrogen-vacancy centre spin decoherence for just one setting of parameters. The holographic principle is applicable for any finite systems of fermions and spins. Because the system size can be arbitrarily large, the physics in systems that approach the thermodynamic limit can be captured. For bosons, however, the validity of theory is verified only in some special cases.

## Results

### Holography of partition function

**Figure 1:**
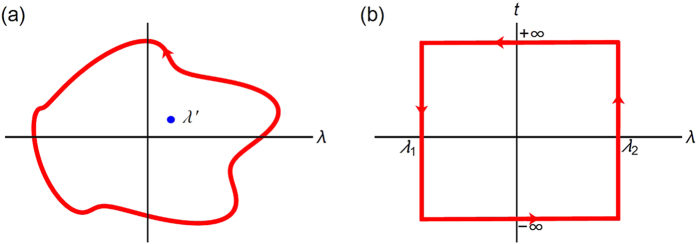
Holography of partition function in the complex plane of a physical parameter. (**a**) Integration contour in the complex plane of *λ*. The red solid curve is the integration contour and *λ*′ is a point inside the analytic domain. (**b**) A rectangular integration contour in the complex plane of a physical parameter *λ* with *t* being the imaginary part. Two vertical straight lines are parallel to the *t*-axis, with real parts being *λ*_1_ and *λ*_2_, and imaginary parts extended from −∞ to +∞. The other two segments are parallel to the real axis with imaginary parts at −∞ or +∞.

The thermodynamic holography states that the partition function of a finite physical system in an area of the complex plane of a physical parameter is uniquely determined by its values along the boundary. This stems from the Cauchy theorem in complex analysis^9^ for analytic functions. The analyticity of partition functions is based on the following Theorem (see Fig. 1a for illustration).

*Theorem 1*. The partition function 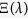 of a finite physical system is analytic in the whole complex plane of a physical parameter *λ*.

The proof is based on the fact that the partition function of a finite system can be expanded into Taylor’s series in terms of the Hamiltonian and the Taylor’s series, being polynomial functions of the parameter *λ*, are all analytic and converge uniformly (see details in Methods). Here by “finite physical system” we mean that the system has a finite number of discrete basis states. For a finite (but arbitrarily large) system of spins and fermions on lattices, the partition function is analytic in the whole complex plane of physical parameters. For bosons, the partition function can be non-analytical in certain regions^13^,^14^ (e.g., the partition functions of free bosons have singularities along the imaginary axis of frequencies, and for coupled bosons the system can become unstable when the coupling is too strong). The theorem can be generalized to infinite-dimensional systems that satisfy certain conditions (see Methods). However, infinite systems in general cases still need further study.

Here comes the main result of this paper. According to Cauchy theorem and Theorem 1, the partition function satisfies


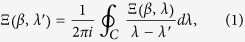


where *λ*′ is a complex parameter enclosed by the integration contour *C* in the complex plane of *λ* where the partition function is analytic (see Fig. 1a). A convenient choice of the boundary (the integration contour) can be a rectangular path (see Fig. 1b) that consists of two straight lines perpendicular to the real axis, whose real parts 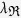 are respectively *λ*_1_ and *λ*_2_, and two segments parallel to the real axis, whose imaginary parts are respectively −∞ and +∞. The contributions from the two segments at infinity vanish based on the inequality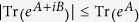 for finite-dimensional Hermitian operators *A* and *B* (see Lemma 1 in Methods for details). Thus we have the following theorem.

*Theorem 2*. The partition function of a finite physical system along two vertical lines in the complex plane of a physical parameter where the partition function is analytic uniquely determines the partition function at any complex parameter between these two vertical lines, that is,


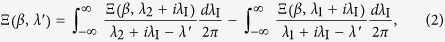


for 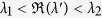.

Theorem 2 can be simplified by introducing a constant *M*_−_ less than the minimum eigenvalue of ∂_*λ*_*H* (which is just *H*_I_ for *H* = *H*_0_ + *λH*_I_) to ensure 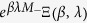 vanishes as 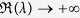. That leads to

*Corollary 1*. If the partition function is analytic on the half complex plane 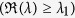 of a physical parameter, the partition function along this line uniquely determines the partition function on that half complex plane, that is,


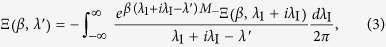


for any complex parameter *λ*′ satisfying 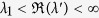.

Similarly, by introducing a constant *M*_+_ greater than the maximum eigenvalue of ∂_*λ*_*H* to ensure 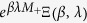 vanishes as 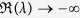, we have

*Corollary 2*. If the partition function is analytic on the half complex plane 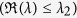 of a physical parameter, the partition function along this line uniquely determines the partition function on that half complex plane, that is,


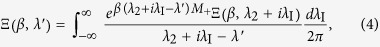


for any complex parameter *λ*′ satisfying 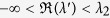.

Corollaries 1 and 2 are particularly usefully for boson systems, where the partition functions may not be analytic on the whole complex plane of a parameter. For example, the partition function of free bosons has singularity points along the imaginary axis.

Equations (2, 3, 4) establish the holographic principle for partition functions. In practical application, one can make use of the partition functions of weakly interacting systems, which can be easy to calculate, to derive the partition functions of strongly interacting systems, which are usually difficult to calculate since the perturbation method may fail in absence of small parameters.

### Possible experimental realization of thermodynamic holography

Recently our group discovered that the partition function of a system with a complex parameter is equivalent to the quantum coherence of a probe spin coupled to the system^10^,^11^,^12^. One can use a probe spin-1/2 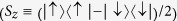 coupled to a system (bath) with bath Hamiltonian *H*(*λ*) and probe-bath interaction 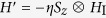, where *η* is a small coupling constant and *H*_I_ = ∂_*λ*_*H*. If the probe spin is initialized in the superposition state 
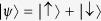
 and the system in the thermal equilibrium with density matrix 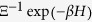, the quantum coherence of the probe spin is quantified by the spin polarization 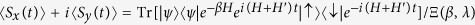. When [*H*, *H*_I_] = 0, the probe spin coherence has an intriguing form as^10^


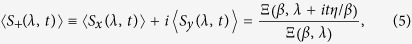


which is equivalent to the partition function with a complex parameter, *λ* + *itη*/*β*. Now the evolution time serves as the imaginary part of the physical parameter. Recently, Lee-Yang zeros have been observed via such a measurement^12^. In more general cases 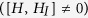, it is possible to engineer the probe-bath interaction so that the probe spin coherence still has the form in equation (5) 11. In terms of the probe spin coherence, equation (2) can be rewritten as


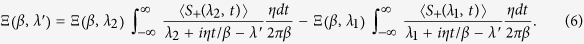


Similarly, equation (3) can be rewritten as


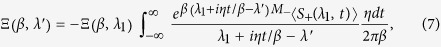


and equation (4) as


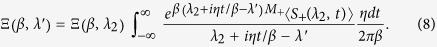


Note that the probe spin coherence resembles the form of quantum quenches^15^. Therefore the quantum quench dynamics may also be studied using the thermodynamic holography.

Equations (6, 7, 8) establish an experimentally implementable holographic approach to thermodynamics. For an arbitrary real parameter *λ*′, we can determine the free energy 
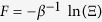
 by


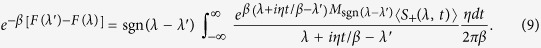


Thus we can extract the full thermodynamic properties of the system from the probe spin coherence measurement for just a single value of the physical parameter. Note that previously the free energy difference (Δ*F*) has been related to the work (Δ*W*) in a non-equilibrium physical process by the Jarzynski equality 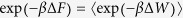
16. The Jarzynski equality is particularly useful for determining free energy differences for small thermodynamic systems such as quantum engines and biomolecular systems^17^,^18^,^19^. Using the thermodynamic holography and the Jarzynski equality, we establish a general relation between the probe spin coherence, the work done on small systems, and the free energy changes. This general relation is indeed the foundation of two recent proposals for experimental measurement of the characteristic function of the work distributions^20^,^21^, which plays a central role in the fluctuation relations in quantum quenches^15^ and more generally in non-equilibrium thermodynamics^22^. The power of the thermodynamic holography is that one can obtain free energy change for any parameters using the probe spin coherence measurement for just one value of the parameter instead of quenching the system to various parameters^17^,^18^,^19^.

The thermodynamic holography can also be used to determine the probe spin coherence for an arbitrary parameter by the coherence measurement for just one value of the parameter. Choosing a complex parameter *λ*′ + *it*′*η*/*β*, we have the probe spin coherence


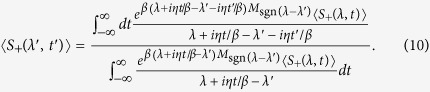


The holographic method can be further simplified in many cases. Since the partition function, 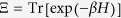, is the sum of exponential function of the Hamiltonian, the probe spin coherence would be a periodic function of time if the energy level differences of a system are quantized in some unit, such as in the spin Ising models^10^. Then in such cases one does not need to measure the probe spin coherence as a function of time from −∞ to +∞. Instead, the probe spin coherence in one period of time, from 0 to 2*π*/*η*, is sufficient to produce the full information of the partition function. In such cases, equation (9) becomes


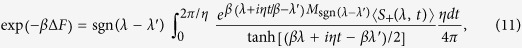


and equation (10) becomes


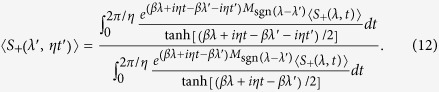


## Discussion

### Thermodynamic holography of a mechanical resonator coupled to a probe spin

**Figure 2:**
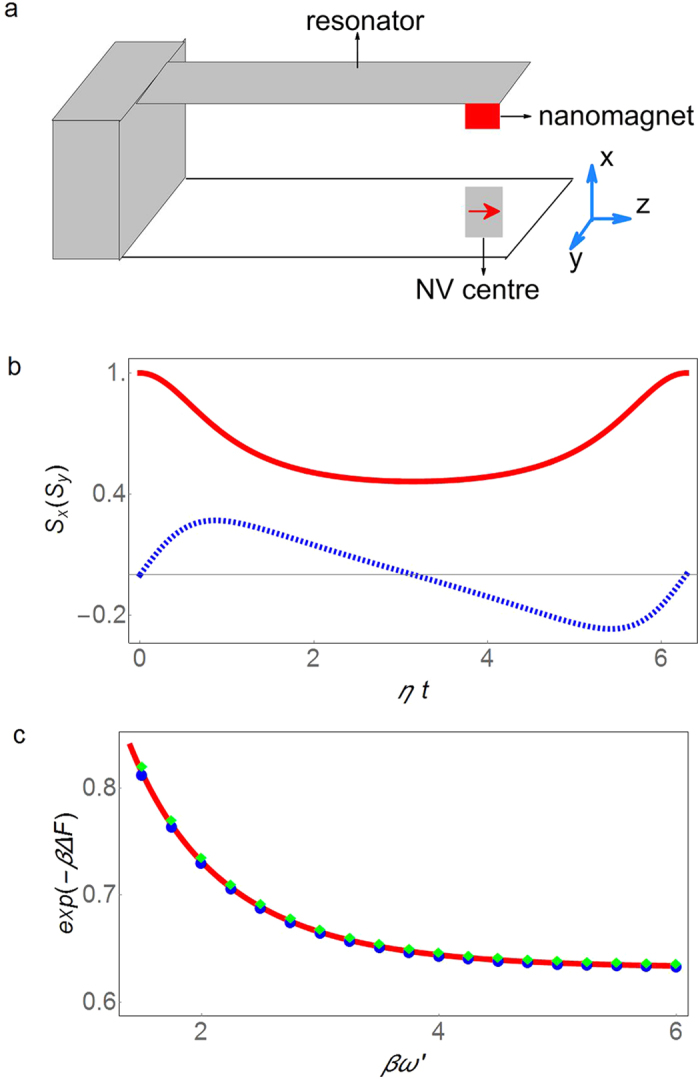
Extracting free energy difference of an oscillator from the probe spin coherence measurement. (**a**) Schematic plot of an NV centre coupled to a mechanical resonator. The mechanical resonator has a magnetic tip attached at the end. An NV centre is placed right under the magnetic tip. The oscillation of the mechanical resonator generates a time-dependent magnetic field on the NV centre spin. (**b**) The spin coherence of the NV center as a function of scaled time, *ηt* with 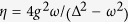, at temperature *βω* =1. The red solid line presents *S*_*x*_ and the dashed blue line shows *S*_*y*_. (**c**) The free energy difference for the mechanical resonator obtained from the probe spin coherence. The solid red line is the direct solution. The symbols are obtained by the holographic method using spin decoherence at *βω* =1. For the green diamonds (blue dots), 63 (157) evenly spaced data points in one period of time have been used in the numerical integration.

To demonstrate the idea of thermodynamic holography, we study an experimentally realizable system as the model example, namely, a nitrogen-vacancy (NV) centre spin coupled to a nano-mechanical resonator^23^,^24^ (see Fig. 2a). This model may also be realized in a superconducting resonator^25^. The NV centre has a spin triplet ground state (*S* = 1) with a large zero field splitting Δ = 2.87 GHz. The mechanical resonator, with frequency *ω* ~ 3 GHz, is assumed to have a magnetic tip. The NV centre is placed right under the tip. The oscillation of the mechanical resonator generates a time dependent magnetic field on the NV centre spin with an interaction Hamiltonian 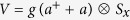. Under realistic conditions, the coupling *g* can reach 1 MHz for a magnetic tip with size of 100 nm and an NV centre located about 25 nm under the tip^26^. The Hamiltonian of coupled mechanical resonator and the NV center is 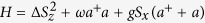. We make use of the spin states 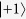 and 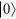 as a probe and define 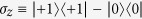 and the corresponding spin flip operators 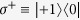 and 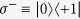. Since the coupling 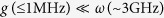 & Δ(~2.87 GHz), the perturbation theory (see Methods for details) gives an effective Hamiltonian


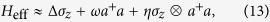


with *η* = 4*g*^2^*ω*/(Δ^2^ − *ω*^2^). The NV center acts as a probe spin and the mechanical resonator as a system (bath). The partition function 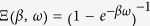 of the oscillator has an infinite number of singularity points *ω* = *i*2*nπ*/*β* (where *n* is an arbitrary integer) along the imaginary axis of the frequency. But the partition function is analytic on the half complex plane of frequency with positive real part (See Methods). Hence the holographic principle applies. We shall demonstrate that the free energy difference can be extracted from the probe spin coherence measurement of the NV center. We assume that the NV center is initialized in the superposition state 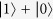 and the mechanical resonator in the thermal equilibrium. Note that in the current case the probe spin coherence is a periodic function of time since the energy levels of the oscillator are equally spaced. So the probe spin coherence in one period of time, from 0 to 2*π*/*η*, is sufficient to yield the full information of the partition function. The measurement time of the NV center spin coherence is ~2*π*/*η* ≈ 100 *μs*, which is within the spin coherence time of an NV centre in an isotopically purified diamond^27^.

Figure 2b presents the real (red solid line) and imaginary (blue dashed line) parts of the NV centre spin coherence as functions of time for the resonator at temperature 150 mK (*βω* = 1). From the spin coherence we can obtain the free energy (relative to the value at *βω* = 1) for arbitrary *βω*. Figure 2c shows the exponentiated free energy difference (green squares and blue dots) constructed via equation (11) from the NV center spin coherence shown in Fig. 2b. The green squares are obtained with numerical integration of 63 evenly separated data points of the NV center spin coherence during one period of evolution at *βω* = 1. The error in the numerical integration is 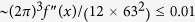, where *f*″(*x*) is the second order derivative of the integrand and 0 ≤ *x* ≤ 2*π*. The blue dots are obtained by numerical integration of 157 evenly separated data points with numerical error 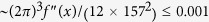. The results obtained by the holographic method agree with the direct calculation of the free energy (the solid red line in Fig. 2c) within the numerical errors.

**Figure 3:**
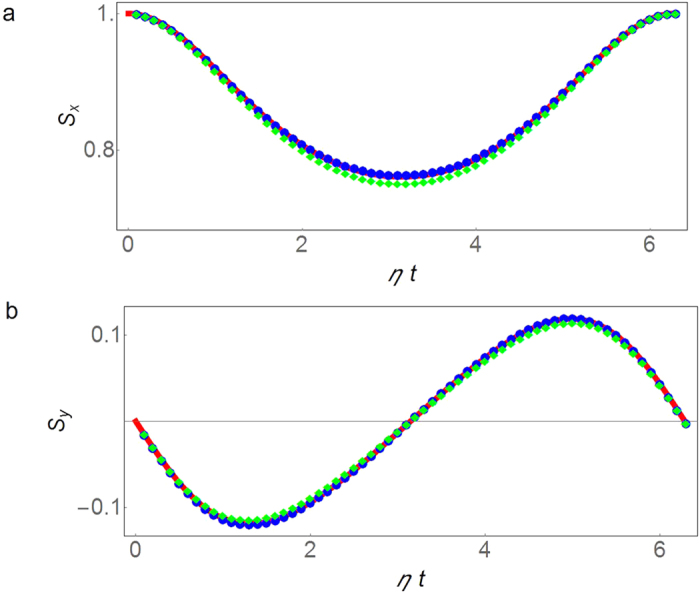
Probe spin coherence by thermodynamic holography. (**a**) The real part of the probe spin coherence at *βω* = 2 as a function of scaled time, *ηt* with 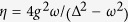. The symbols are obtained by the holographic method using spin decoherence at *βω*  = 1. For the green diamonds (blue dots), 63 (157) evenly spaced data points in one period of time have been used in the numerical integration. (**b**) The same as (**a**) but for the imaginary part of the spin coherence.

The holographic approach can also be used to determine the probe spin coherence for arbitrary *βω*. The real and imaginary parts of the constructed spin coherence for *βω* = 2 as functions of time are presented respectively in Fig. 3a,b. The green squares are obtained by equation (12) with numerical integration of 63 evenly spaced data points of the NV center spin coherence at *βω* = 1 during one period of time. The error in the numerical integration is 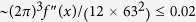. The blue dots are obtained by numerical integration of 157 evenly spaced data points with numerical error 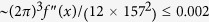. The results agree with the direct solution within numerical errors.

## Summary

In this work we have established the concept of thermodynamic holography. We prove that the partition function of a finite physical system in an area of the complex plane of a physical parameter is uniquely determined by its values along the boundary. Since the partition function with a complex parameter is equivalent to the probe spin coherence, one can experimentally implement the thermodynamic holography through probe spin coherence measurement for just one physical parameter. Thermodynamic holography may have applications in studying thermodynamics of nano-scale systems (such as molecule engines^28^, nano-generators^29^ and macromolecules^17^,^30^) and provide a new approach to many-body physics.

## Methods

### Proof of Theorem 1

The system has a finite number (*K*) of basis states. We define a series of functions 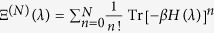, which are sums of a finite number of polynomial functions of *λ*. Obviously, all these functions are analytic in the whole complex plane of *λ*. Considering the parameter in the range 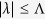, for an arbitrarily small quantity *ε*, there exists an integer *N*_*ε*_ such that 
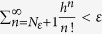
, where *h* is the maximum of 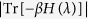 for 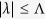. Therefore 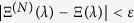 for *N* > *N*_*ε*_, i.e., the function series uniformly converge to the partition function in the parameter range 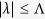. According to the uniform convergence theorem of analytic functions^9^, the partition function is analytic for 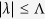. Since 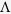 can be chosen to be arbitrarily large, Theorem 1 is proved.

### A trace inequality

*Lemma 1*. Two Hermitian operators *A* and *B* satisfy the trace inequality 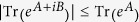 for a system that has a finite number of basis states, or for a system that has an infinite number of discrete basis states if *B*, [*A*, *B*], [*A*, [*A*, *B*]], … commute with each other.

*Proof*. For a system with a finite number of basis states, both *A* and *B* are finite dimensional matrices. By Hölder’s inequality for finite-dimensional matrices^31^,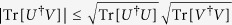 for operators *U* and *V*, we have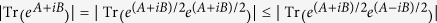. Then according to Bernstein inequality^32^, 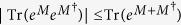 for an operator *M*, we have 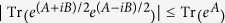 and hence 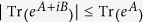.

We now consider infinite-dimensional systems. We define 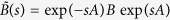. If *B*, [*A*, *B*], [*A*, [*A*, *B*]], … commute with each other, 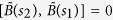 for arbitrary real numbers *s*_1_ and *s*_2_. Thus we have^33^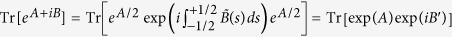, where 
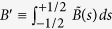
 is Hermitian. By Cauchy Schwarz inequality^31^, we have 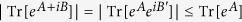.

Thus Lemma 1 is proved.

For more general cases of infinite-dimensional operators, however, we have not been able to prove the trace inequality 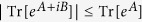. It needs further study.

### Generalization of Theorem 1 to certain infinite-dimensional systems

For a physical system with an infinite number of basis states (such as bosons), we assume that the basis states are discrete (countable), which is always possible since we can confine the system using a sufficiently large box.

We can generalize Theorem 1 to infinite-dimensional Hamiltonians that satisfy the specified condition for Lemma 1 (i.e., *H*_I_, [*H*, *H*_I_], [*H*, [*H*, *H*_I_]], … commute with each other). We choose the eigenstates of *H* as the discrete basis states denoted by 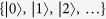 in the ascending order of eigenvalues and define a truncated series of partition functions 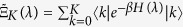 in a finite subspace 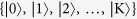, which are of course analytic functions of *λ*. If we can prove that these truncated partition functions 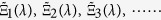 approach to 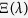 uniformly, then according to the uniform convergence theorem of analytic functions^9^, the partition function 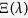 is analytic. If the partition function 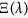 exists in a close segment on the real axis 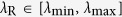, there always exists an integer *K*_*ε*_ for an arbitrary small positive number *ε* such that 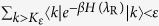 for 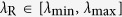. According to Lemma 1, 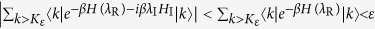 if *H*_I_, [*H*, *H*_I_], [*H*, [*H*, *H*_I_]], … commute with each other. So the series of analytic functions 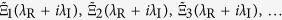 uniformly converge to 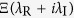 for 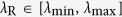. According to the uniform convergence theorem of analytic functions^9^, the partition function 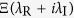 is analytic for 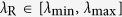. Thus Theorem 1 applies to infinite-dimensional systems if *H*_I_, [*H*, *H*_I_], [*H*, [*H*, *H*_I_]], … commute with each other.

### Proof of Theorem 2

We just need to show 
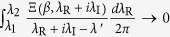
 for 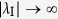. Suffices it to show that 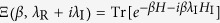 is bounded for 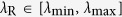. According to Lemma 1, 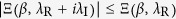. Since 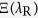 is bounded for 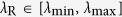, 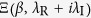 is bounded. Therefore Theorem 2 is proved.

### Generalization to Hamiltonians that are general analytic functions of parameters

For Hamiltonians that are general analytic functions of the parameters, 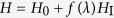, then 
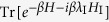
 is be replaced by 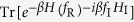, where 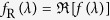 and 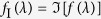. Theorem 1 and 2 and Corollaries 1 and 2 still hold but the specific forms of Corollaries I and II depend on the specific forms of the Hamiltonians when the parameter is extended to positive and negative infinity.

### Derivation of the effective Hamiltonian of a coupled spin-oscillator system

The coupling Hamiltonian for the pseudo spin and the oscillator is 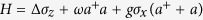. Since 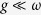, Δ, we can treat 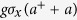 as a perturbation. A unitary transformation defined by 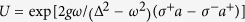 leads to 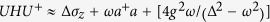 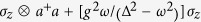. The last term can be dropped since it is only a small correction to the Zeeman energy of the spin. We therefore obtain equation (13).

### Numerical method

In determining the free energies of the mechanical oscillator from the probe spin coherence through thermodynamic holography, we took equally spaced points of the NV centre spin coherence within one period 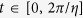 and then carried out numerical integrations by the trapezoidal rule.

## Additional Information

**How to cite this article**: Wei, B.-B. *et al.* Thermodynamic holography. *Sci. Rep.* **5**, 15077; doi: 10.1038/srep15077 (2015).
